# Non-coding RNAs change their expression profile after Retinoid induced differentiation of the promyelocytic cell line NB4

**DOI:** 10.1186/1756-0500-3-24

**Published:** 2010-01-27

**Authors:** Annalisa Rossi, Oscar F D'Urso, Graziana Gatto, Palmiro Poltronieri, Manuela Ferracin, Paolo Remondelli, Massimo Negrini, Maria G Caporaso, Stefano Bonatti, Massimo Mallardo

**Affiliations:** 1 Department of Biochemistry and Medical Biotechnologies, University of Naples Federico II, Via S. Pansini 5, Napoli, Italy; 2 Biotecgen, Ecotekne, via prov.le Monteroni, Lecce, Italy; 3 ISPA-CNR Ecotekne, Lecce, Italy; 4 Department of Experimental and Diagnostic Medicine, Interdepartmental Center for Cancer Research, University of Ferrara, Ferrara 44100, Italy; 5 Department of Pharmaceutical Sciences, University of Salerno, via Ponte Don Melillo, Fisciano SA, Italy

## Abstract

**Background:**

The importance of non-coding RNAs (ncRNAs) as fine regulators of eukaryotic gene expression has emerged by several studies focusing on microRNAs (miRNAs). miRNAs represent a newly discovered family of non coding-RNAs. They are thought to be crucial players of human hematopoiesis and related tumorigenesis and to represent a potential tool to detect the early stages of cancer. More recently, the expression regulation of numerous long ncRNAs has been linked to cell growth, differentiation and cancer although the molecular mechanism of their function is still unknown.

NB4 cells are promyelocytic cells that can be induced to differentiation upon retinoic acid (ATRA) treatment and represent a feasible model to study changes of non coding RNAs expression between cancer cells and their terminally differentiated counterpart.

**Findings:**

we screened, by microarray analysis, the expression of 243 miRNAs and 492 human genes transcribing for putative long ncRNAs different from miRNAs in NB4 cells before and after ATRA induced differentiation. Our data show that 8 miRNAs, and 58 long ncRNAs were deregulated by ATRA induced NB4 differentiation.

**Conclusion:**

our data suggest that ATRA-induced differentiation lead to deregulation of a large number of the ncRNAs that can play regulatory roles in both tumorigenesis and differentiation.

## Background

The past few years have revealed that the genomes of all studied eukaryotes are almost entirely transcribed, generating an enormous number of long and small non-protein-coding RNAs (ncRNAs) [1-5]. In parallel, it is increasingly evident that many of these ncRNAs have regulatory functions.

Considerable work has been done on small regulatory RNAs. By miRNA microarray analysis, several laboratories have performed the miRNA expression profile (miRNome) in cancer patients and found that miRNAs are differentially expressed in normal and tumor tissues [5-7]. These differences are often tumor-specific and, potentially, can be related to diagnosis and prognosis.

In addition to these small ncRNAs, there are thousands of longer transcripts whose functions are still unknown [1,8-10]. Very recently, they have been functionally linked to cancer and cell differentiation. Long ncRNAs, such as H19 and BIC, can exert multiple functions. Indeed, H19 long ncRNA promotes breast cancer cell proliferation through positive control of E2F1 [11] and can be processed into smaller RNA sequences having the feature of miRNA [12]. The longer transcript BIC originates miR-155, shown to be important in the hematopoietic function [13] as well as in the homeostasis and function of the immune system [14]. These examples confirm the idea that also long ncRNAs can play a role in cell transformation and differentiation.

NB4 is a promyelocytic cell line derived from the peripheral blood of a M3 subtype Acute Promyelocytic Leukemia (APL) patient [15]. Treatment with all-trans-retinoic acid (ATRA) is able to revert the dominant-negative effect of PML-RARα fusion protein and induce cell differentiation [16]. Therefore NB4 cells represent a feasible model to study changes of ncRNAs expression between cancer cells and their terminally differentiated counterpart. While miRNAs have been implied in NB4 differentiation and tumorigenesis, there is a lack of knowledge about the expression and function of other families of ncRNAs. Fazi and co-workers showed that upregulation of miR-223 lead to repression of NF1-A translation and this is relevant in the early stages of myeloid differentiation [17]. Garzon et al. [18] showed by microarray analysis, that several miRNAs are subjected to changes in the expression profile during ATRA induced myeloid differentiation of promyelocytic cells.

In this work we used Ribochips to verify the expression profile of 243 miRNAs and 492 long ncRNAs, during ATRA induced differentiation. We found 8 miRNAs and 58 long ncRNAs whose expression levels changed during differentiation. Our work indicates that a wide variety of ncRNAs is regulated during differentiation and suggests their involvement in cell differentiation and tumorigenesis.

## Methods

### Chemicals

All chemicals were purchased from Sigma. All Trans Retinoic Acid (ATRA) was dissolved in ethanol at a concentration of 1 mM stock solution and used at 0.5 μM.

### Cell culture

Cells were cultured in RPMI 1640 medium supplemented with 10% foetal calf serum (FCS), 2 mM L-Glutamine in 5% CO_2 _atmosphere. Both NB4 and HL60 cell lines (2 × 10^5 ^cells/ml) were treated with 0.5 μM ATRA. Differentiation was evaluated following the expression of the antigen CD11c and CD11b by cytofluorimetry. Cell viability was assayed by cytofluorimetry evaluating the incorporation of propidium iodine into the dead cells.

### RNA extraction

At various time after ATRA treatment, 1 × 10^7 ^cells were collected and washed in PBS. Total RNAs were extracted using TRIzol RNA extraction system (Invitrogene) according to the manufacturer's instruction. The integrity of the extracted RNA was assayed by 1% agarose denaturing gel electrophoresis. The ribosomal RNA 28S and 18S were considered for assessing the integrity of the total RNA.

### Northern blot analysis

All RNA samples were dissolved in loading buffer (0.05% bromophenol blue, 0.05% cyanol xilene, 5% Ficoll (type 400), 80% formamide and 7 M urea), boiled for 5' and loaded onto 15% polyacrylamide denaturing gel (15% acrylamyde-bis-acrylamide 19/1, 45 mM Tris, 45 mM boric acid, 1 mM EDTA pH 8, 7 M urea, 0.01% TEMED and 0.1% Ammonium persulphate). Samples were resolved by electrophoresis (90' at 150 V) and transferred on nylon membrane (Hybond N^+^, Amersham) by capillary blot. The membranes, equilibrated in 1 M NaCl, were pre-hybridized in 6× SSC, 5× Denhart's (0.1% Ficoll, 0.1% polyvinyl pirrolydone, 0.1% Bovine Serum Albumin), 100 μg/ml of sheared salmon sperm dsDNA (Ambion) at 42°C for 2 hr. 1 × 10^6 ^CPM/ml of [γ^32^P-ATP] radiolabelled probe was added and the hybridization carried over night at 42°C. The membranes were washed twice in 2× SSC at 42°C for 30' and exposed either by autoradiography or by phosphorimage screen (Amersham). Quantification of the related signals was performed by image-scanning or Image-J software analysis.

### Real Time PCR

Primers were designed (sequences are available in the additional files) for the following genes randomly selected between those found significantly expressed in cancer cell lines: THC1225071, AK021516, NR_002196_H19, AK022994, AK092435, AK128567, AK027352, AK097934, AL122122, CR593144, THC1242508. The microarray expression data was validated by real-time quantitative PCR using the Platinum SYBR Green qPCR SuperMix containing uracil DNA glycosylase (UDG) (Invitrogen) according to manufacturer instructions after cDNA synthesis with SuperScript III First-Strand Synthesis System for RT-PCR. Briefly 44 μl Master mix containing all of the reaction components except the primers was dispensed into a 96-well real-time PCR plate (Applied Biosystems) using a 8-channel pipette. The master mix contained 1.5 U Platinum *Taq *DNA polymerase, 20 mM Tris-HCl (pH 8.4), 50 mM KCl, 3 mM MgCl_2_, 200 μM dNTP, 1 μl UDG and water to 44 μl. All the PCR reagents were from the SYBR green core reagent kit (Invitrogen). A 10 μM solution of each pair of primers was stored in 8-well PCR strip tubes. Each primer (1 μl) was dispensed into duplicate wells of the 96-well plate using the 8-channel pipette. Real-time PCR was performed on an Applied Biosystems 7500 real-time PCR instrument equipped with a 96-well reaction block. PCR was performed for 2 min at 50°C and 2 min at 95°C for one cycle. Then 95°C for 15 s, 64°C for 30 s and 72°C for 35 s for 45 cycles followed by the thermal denaturation protocol. Two normalised human pooled tissue total RNA (Stratagene) were used for comparison. Briefly, we used human normal tissue from brain (stratagene cat. N. 540006) and from testis (stratagene cat. N. 540050). The two samples were pooled and used as control RNA. Relative quantifications were performed by determining the threshold value as by ABI. All data were normalised to actin content. Comparative non-coding RNA expression analysis in treated vs. untreated cells was performed as follows: the ΔCt value was calculated by subtracting the Ct value of the non-coding RNA from the Ct value of actin. The ΔΔCt value was calculated by subtracting the ΔCt value for the untreated cell from to the ΔCt value of the treated cells. Fold of enrichment values were determined according to the following formula: 2^-ΔΔCT^. In additional files 1 and 2 the comparative non-coding RNA expression analysis in cancer cells vs. human normal tissue (control in the graphs) was performed as follows: the ΔCt value was calculated by subtracting the Ct value of the non-coding RNA from the Ct value of actin. The ΔΔCt value was calculated by subtracting the ΔCt value for the human normal tissue from to the ΔCt value of the cancer cells Fold of enrichment values were determined according to the following formula: 2^-ΔΔCT^.

Details about the long ncRNAs Array Fabrication, Fluorescent Labeling, Hybridization, Image Analysis and Data Collection and Western blot are described in the additional file 3.

## Results and Discussion

### miRNAs expression profile in ATRA treated NB4 cells

**Figure 1:**
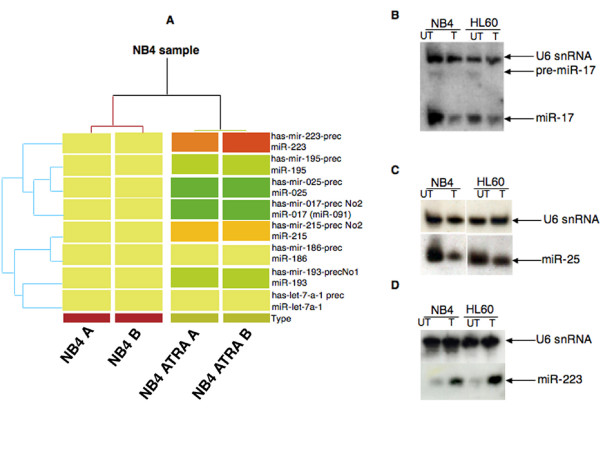
**Cluster analysis of treated and untreated NB4 cell lines using the 8 human miRNAs differentially expressed and Northern blot validation of miR-223, miR-17 and miR-25**. A) Cluster analysis, made only for the human miRNAs, reveals that the 8 miRNAs changed their expression after treatment. In fact, red color means that the expression of the miR is higher in NB4 cell line treated with ATRA while green color means that the expression is lower. Yellow means no variation. B, C, D) 15 μg of total RNA was loaded in each lane from two independent experiments. The membranes were probed as indicated on the side: B) miR- 17 probe and U6 snRNA probe; C) miR-25 probe and U6 snRNA probe; D) miR- 223 probe and U6 snRNA probe. Note that for the miR-17 is visualized also the precursor (prec. miR-17). T = 6 days of 0.5 mM ATRA treatment, UT = untreated.

We compared the miRNA expression profile of terminally differentiated neutrophils, NB4 ATRA treated [additional file 4], and untreated NB4 cells. In order to increase the significance of the microarray analysis we hybridised the RNA derived from two independent experiments. The statistical analysis (Welch t-test and SAM) identified 8 miRNAs differentially expressed (Table 1), 72 miRNAs equally expressed before and after treatment [additional file 5] and 163 miRNAs expressed within the background, hence considered as unexpressed. In detail, the expression of **miR-186**, **miR-215 **and **miR-223 **resulted upregulated in ATRA differentiated cells, while the expression of miR-17-5p, miR-25, **miR-193**, **miR-195**, and **let-7a **resulted downregulated (the miRNAs bolded were already reported as deregulated by ATRA in differentiated NB4 cells in refs. 17 and 18). The cluster analysis, derived from two independent experiments for the 8 human miRNAs is shown in figure 1A.

**Table 1:** miRNAs with altered expression after ATRA induced differentation in NB4 cells by microarray analysis.

Name	P-value	NB4 expression	NB4 + ATRA expression	*Map*	TvsUT	Validation	Densitometry
**hsa-mir-017-precNo2**	0,0447	2,944	1,388	13q31	Down	down	6.0 fold
**hsa-mir-186-prec**	0,0447	0,864	0,891	1p31	Up	up	0.5 fold
**hsa-mir-193-precNo1**	0,0385	1,031	0,847	17q11.2	Down	down	1.2 fold
**hsa-mir-195-prec**	0,0373	2,618	1,954	17p13	Down	down	0.8 fold
**hsa-mir-025-prec**	0,0351	7,56	3,157	7q22	Down	down	4.1 fold
**hsa-let-7a-1-prec**	0,0243	0,962	0,889	9q22.2	Down	up	3.2 fold
**hsa-mir-215-precNo2**	0,0104	0,691	0,792	1q41	Up	up	0.5 fold
**hsa-mir-223-prec**	0.0232	8.68	17.08	Xq12-13.3	Up	up	10.6 fold

In the table are showed for each miRNA the name (hsa = human miRNAs), the P-value, the normalization of two independent experiment (NB4 = untreated; NB4-ATRA = treated); the locus map (map); the expression in treated versus untreated cells (T vs UT) and the expression (T vs UT) after northen blot validation (validation: up = upregulation; down = downregulation); the densitometry shows the average of three independent experiments.

### miR-17-5p and miR-25 are downregulated in NB4 cells differentiated upon ATRA treatment

The microarray data indicated a strong downregulation of miR-17 and miR-25 in the differentiated phenotype (Figure 1B and 1C, NB4 lanes UT vs. T), two miRNAs not yet described as deregulated during ATRA induced differentiation of NB4 cells. Thus we focalized our analysis on them. We confirmed the microarray data by Northern blot, using as DNA probes the antisense mature sequence of both deregulated miRNA. All northern blots were normalized against U6 snRNA. The densitometric analysis, carried out on three independent experiments, showed that in ATRA treated NB4 cells the expression of miR-17 decreased about 6 fold while the expression of miR-25 decreased about 4 fold. These results were confirmed also in HL60 cells, (Figure 1B and 1C, HL60 lanes UT vs. T) another promyelocytic cell line inducible to granulocyte differentiation by retinoids. In both cell lines we observed a proportional correlation between the amount of the mature miR-17 and its precursor suggesting that this miRNA is mainly regulated at transcriptional level during myeloid differentiation. As positive control we checked for the expression of miR-223, upregulated during myeloid differentiation [17]. As expected, miR-223 was found expressed 10 fold higher after ATRA induced differentiation (Figure 1D) in both NB4 and HL60 cells.

By *in silico *analysis we found that both miR-25 and miR-17 could have as putative targets E2F1 and E2F4, thus, they could synergistically act in the control of promyelocytic proliferation and differentiation. To test our hypothesis we analyzed the expression level of E2F1 and E2F4 proteins in untreated and ATRA differentiated NB4 cells. As shown in additional file 6, both the proteins as well as the two miRNAs were downregulated by ATRA differentiation. These results would suggest that miR-17-5-p and miR-25 do not target E2F transcription factors, in NB4 cells from Acute Promyelocytic Leukemia. For this reasons, further studies on the identification of genes possessing miR-17-5p or miR-25 complementary sequences are required.

The relevance of miR-223 upregulation during the early stages of myeloid differentiation has been described [17]. We observed a ten-fold upregulation of miR-223 expression in our system, when the CD11^+ ^positive cells are more than 90%, hence terminally differentiated [additional file 4]. Thus, miR-233 may represent not only an early factor required for myeloid differentiation but it could play an additional important role in the maintenance of the differentiated phenotype.

### Long non-protein coding RNAs are deregulated during ATRA differentiation of NB4 cells

**Figure 2:**
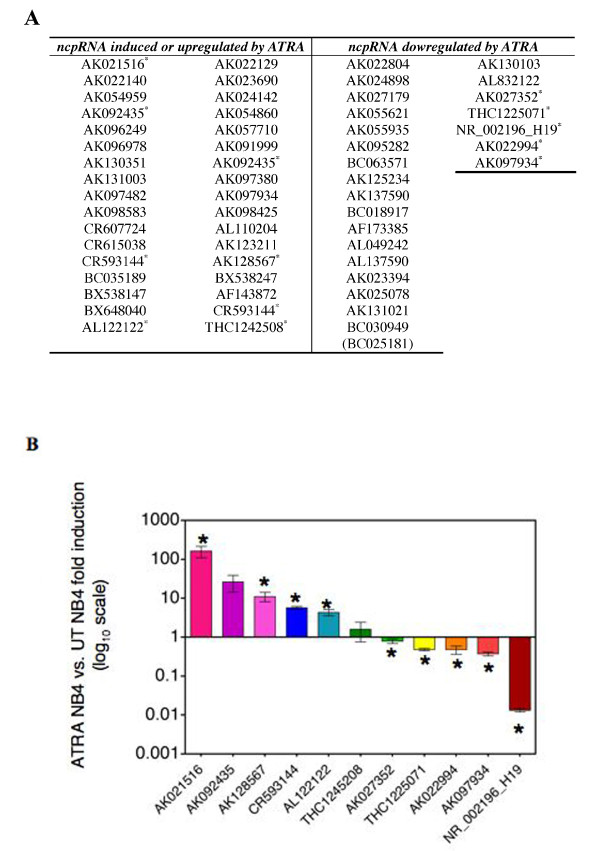
**Long non-protein coding RNAs are deregulated during ATRA differentiation of NB4 cells**. A) In the table are showed the 58 ncRNAs found deregulated in ATRA treated NB4 cells. Asterisks indicate the ncRNAs validate by quantitative real-time PCR. B) Real time confirmed the microarray data. The experiments, performed three times, are shown as fold induction of ATRA NB4 vs. untreated NB4 (UT). The bars indicates ± SEM. Statistical analysis was performed with Prism v4.0 (GraphPad software); asterisk indicate significant differences between ATRA treated NB4 and UT NB4 according to the two-tailed unpaired Student's t test (*, P < 0.05). The primer sequences, used for the real time PCR are shown in the supplementary file 3.

DNA array hybridisation experiments were performed to compare the expression levels of 492 ncRNA genes in NB4 versus NB4 + ATRA treated cells. Fifty-eight genes were found deregulated (Figure 2A), 144 genes equally expressed in both NB4 phenotypes and 290 expressed within the background, hence considered as unexpressed. Among the 58 deregulated genes eleven randomly chosen were validated by Real Time PCR (Figure 2B). The statistical analysis was performed with Prism v4.0 (GraphPad software).

The variation in the expression of the detected ncRNAs in both NB4 cells phenotypes was analyzed and compared to their expression in several cancer cell lines. In additional file 1 and 2 is shown the Real Time analysis in cancer cell lines of twelve deregulate ncRNAs as an example. This analysis allowed us to validate the ontogeny groups assigned through differential expression in proliferating versus differentiated NB4 cells.

The group of deregulated genes, represented by AK092435, AK130351, AK131003, BC035189, AK023690, AK097380, AK098425 AK128567 and AL110204 was found upregulated by ATRA-induced differentiation in NB4 cells (Figure 2A). All these genes, highly expressed in cancer cells (data not shown), may therefore be considered as genes not involved in proliferation of NB4 cells, so that their expression could result from the activity of transcription factors regulated by ATRA signalling, as NF-k-B, C/EBP-α, C/EBP-β or C/EBP-ε. A second group of transcripts found overexpressed in cancer cell lines and downregulated in ATRA-differentiated NB4 cells is represented by AK025078 (SLC26A2), AK027179, AK055935, AK125234 and BC030949, a gene potentially involved in the juvenile leukaemia. These genes are potentially linked to proliferation and they could be upregulated through myc and ras signalling pathway, activated in proliferating NB4 cells [19].

AK001558, a gene upregulated in human retinal Muller cells and protecting against oxidative stress [20], was found expressed in cancer cell lines but showed similar expression in both proliferating and differentiated NB4 cells (data not shown). In addition to ncRNAs distributed randomly onto all chromosomes the Ribochip contained 70 transcripts from the chromosome 7, to analyse their expression changes associated to the transcripitonal state of the HOXA locus. Although most of these transcripts did not show significant variations, untreated NB4 cells showed several upregulated genes, as 7p21 (GA 3603), downstream of HOXA gene cluster, while after differentiation we found upregulation of AK026386, in the 7p21 region, as well as GA3749 in 7p13 and AK026119 in 7p14.

Finally, *H19 *is overexpressed in some types of cancers [12] and it was found expressed at highest levels in proliferating NB4 cells (Figure 2B) and in tumor cell line such as the ERBB2-overexpressing SKBR3 cell line [additional files 1 and 2] and the 883KE testis cancer cell line [additional files 1 and 2]. Thus H19 seems to be involved in cell proliferation.

All together these data show that, even if the function of long ncRNAs as tumor suppressors or as oncogenes can be only hypothesized to date, their expression is related to cell physiology and in some case is tumor specific.

In conclusion, this work shows that several ncRNAs are potentially involved in proliferation and ATRA-induced differentiation of NB4 cells. The association of functional characterization of the studied genes with their expression in cancer may provide clues on their ontology class, and possibly on their function in the aetiology of diseases. Moreover, the increasing number of ncRNAs whose expression profile changes accordingly to the cell phenotype (transformed or terminally differentiated) could represent, in the near future, a suitable biomarker to characterize differentiation.

## Competing interests

The authors declare that they have no competing interests.

## Authors' contributions

A.R., G.G. and M.G.C. performed the miRNA experiments; O.F.D and P.P. performed the long ncRNAs experiments and statistical analysis; M.F. and M.N. provided the miRNA microarray statistical analysis; P.R. and S.B. contributed to manuscript preparation; M.M. conceived and supervised this study and contributed to manuscript preparation. All authors read and approved the final manuscript.

## Supplementary Material

Additional file 1 **Expression by Real time PCR of 15 ncRNAs in cancer cell lines**. RT-PCR of ncRNA expression data using the Ribochip in different cancer cell lines, using primer pairs for selected genes AF143872, AK022994, TCAG1963768, AK094435, THC1225071, AL122122, THC1242508, AK128567, AK098425, H19, CR593144, AF143872, AK021516, AK027352, AK097934, AK097482, randomly selected, and significantly expressed in the cancer cell lines SKBR3, MCF-7, HS-578T (Breast cancer); Be[2]C, CCF-STTG1, DAOY1, (Brain cancer); LS174-T, Caco-2, WiDr, SW1116 (Colon cancer), GLC-4, H226 (Lung cancer), 833KE, NT2, Tera-1 (Testis cancer). As control we pooled human normal tissue total RNA from brain and from testis. Click here for file

Additional file 2 **Expression by Real time PCR of 15 ncRNAs in cancer cell lines**. See Additional file 1 Click here for file

Additional file 3 **Additional M&M information**. Materials and Methods for Array Fabrication, Fluorescent Labeling and Hybridization, Image Analysis and Data Collection, Human genome bioinformatic analysis, Microarray analysis, ATRA-induced differentiation of promyelocytic NB4 cells. Western blot analysis and Primers sequences for Real Time validation. Click here for file

Additional file 4 **Representative experiment of ATRA induced differentiation of NB4 cells assayed by FACS analysis**. A and B) CD11^+ ^FACS readout in NB4 untreated (A) or ATRA treated cells (B) using PE-conjugated α-CD11c mAb; C and D) cell viability FACS readout in NB4 untreated (C) or ATRA treated cells (D) using propidium iodine. Click here for file

Additional file 5 **miRNAs equally expressed in both ATRA treated and untreated NB4 cells by microarray analysis**. Hsa = human miRNAs; mmu = miRNAs Click here for file

Additional file 6 **E2F1 and E2F4 proteins level don't correlate with the miR-25 and miR-17 expression in NB4+ ATRA treated cells**. 30 μg of total proteins were extracted at the indicated times from ATRA treated (T) or untreated (UT) NB4 cells and analyzed by western blot using α-E2F1 and α-E2F4 polyclonal antibodies (Santa Cruz). Input proteins were equalized by detecting the endogenous γ-tubulin. Click here for file
