# Supplementary material for: Non-coding RNAs change their expression profile after Retinoid induced differentiation of the promyelocytic cell line NB4
Source: BMC Res Notes. 2010 Jan 27;3:24. doi: 10.1186/1756-0500-3-24 (PMC2843733; doi:10.1186/1756-0500-3-24)
Supplement: Additional file 1 — Expression by Real time PCR of 15 ncRNAs in cancer cell lines. RT-PCR of ncRNA expression data using the Ribochip in different cancer cell lines, using primer pairs for selected genes AF143872, AK022994, TCAG1963768, AK094435, THC1225071, AL122122, THC1242508, AK128567, AK098425, H19, CR593144, AF143872, AK021516, AK027352, AK097934, AK097482, randomly selected, and significantly expressed in the cancer cell lines SKBR3, MCF-7, HS-578T (Breast cancer); Be[2]C, CCF-STTG1, DAOY1, (Brain cancer); LS174-T, Caco-2, WiDr, SW1116 (Colon cancer), GLC-4, H226 (Lung cancer), 833KE, NT2, Tera-1 (Testis cancer). As control we pooled human normal tissue total RNA from brain and from testis. [file 1756-0500-3-24-S1.PDF]

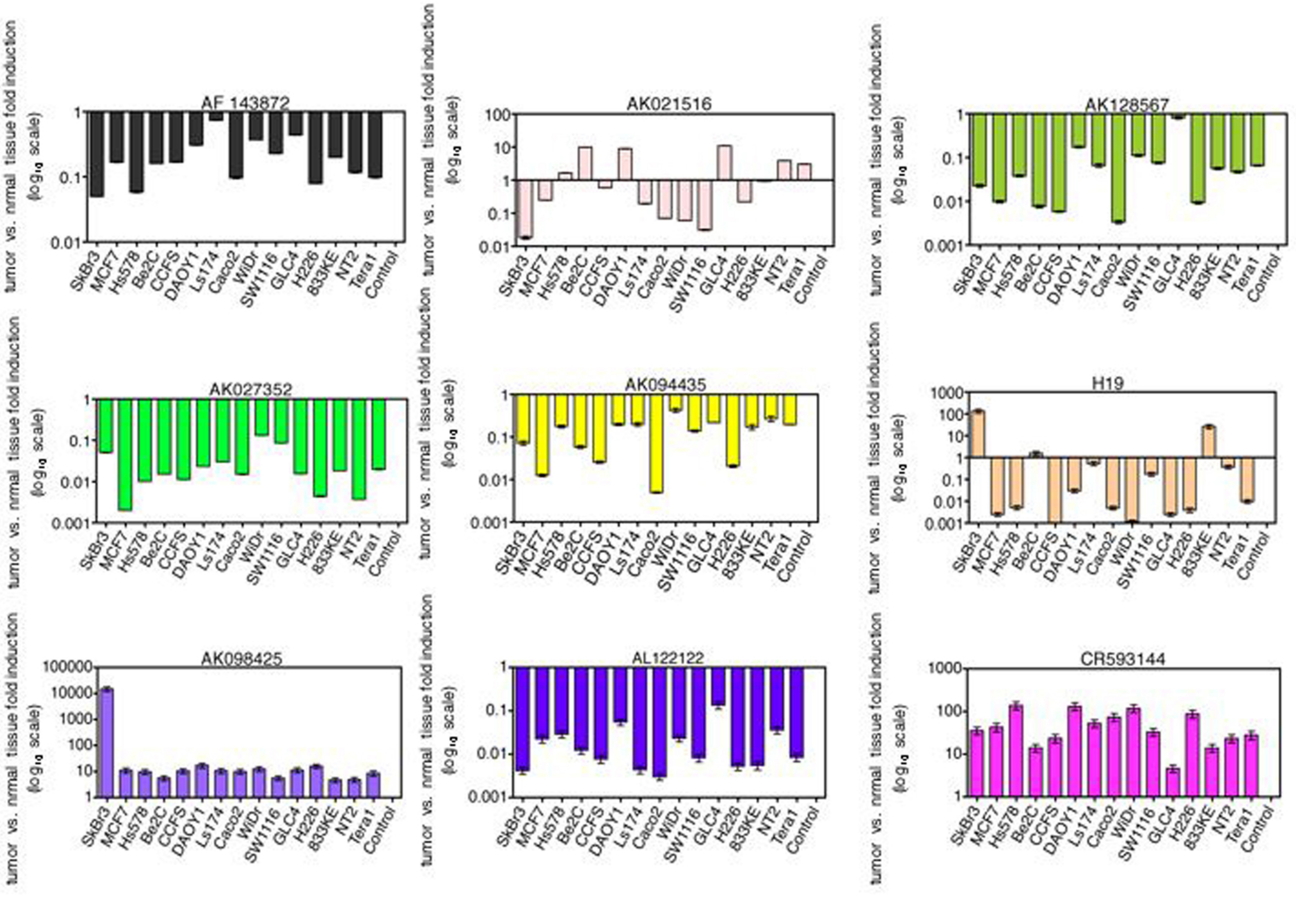
AF 143872
AK021516
AK128567
AK027352
AK094435
H19
AK098425
AL122122
CR593144
tumor vs. nrmal tissue fold induction
(log10 scale)
SkBr3
MCF7
Hs578
Be2C
CCFS
DAOY1
Ls174
Caco2
WiDr
SW1116
GLC4
H226
833KE
NT2
Tera1
Control
100000
10000
1000
100
10
1
0.1
0.01
0.001
