# Supplementary figures and images for: Non-coding RNAs change their expression profile after Retinoid induced differentiation of the promyelocytic cell line NB4
Source: BMC Res Notes. 2010 Jan 27;3:24. doi: 10.1186/1756-0500-3-24 (PMC2843733; doi:10.1186/1756-0500-3-24)

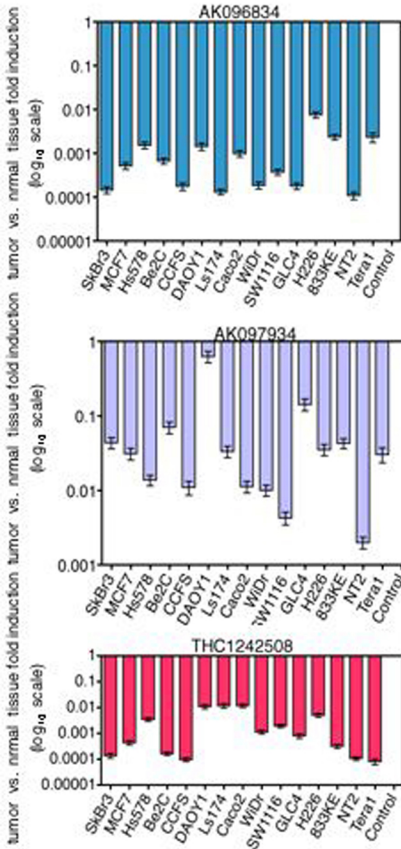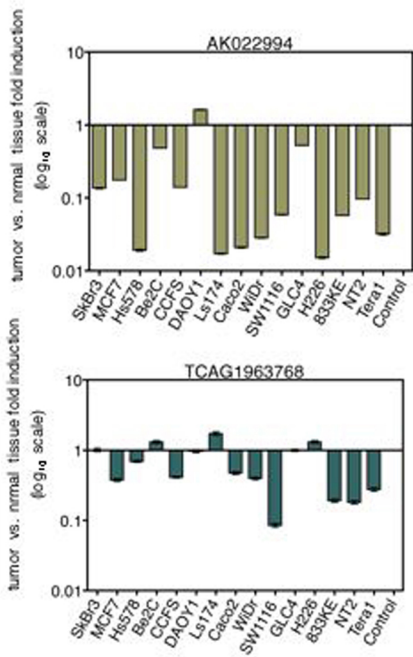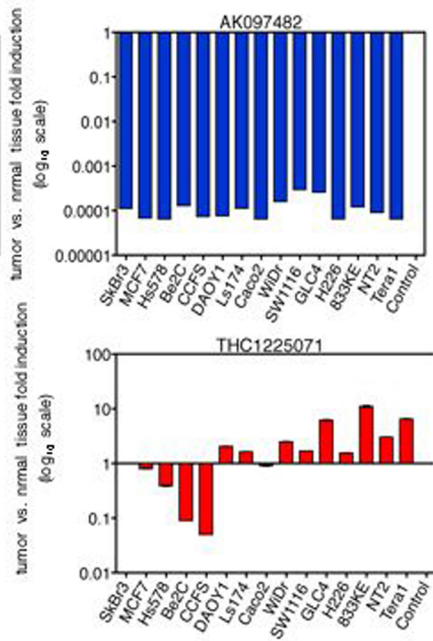

Supplement: Additional file 2 — Expression by Real time PCR of 15 ncRNAs in cancer cell lines. See Additional file 1 [file 1756-0500-3-24-S2.PDF]

**A**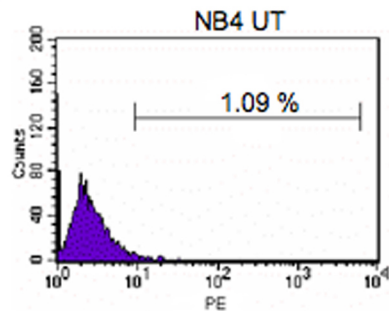**B**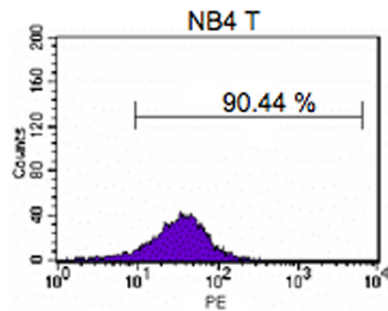

CD11c

**C**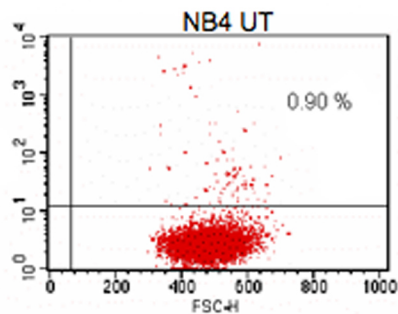**D**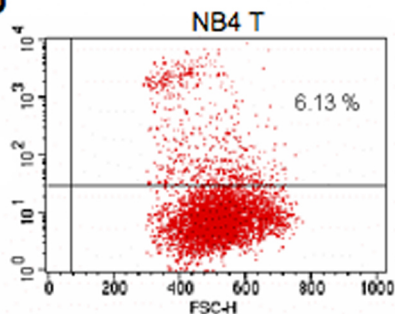

Supplement: Additional file 4 — Representative experiment of ATRA induced differentiation of NB4 cells assayed by FACS analysis. A and B) CD11+ FACS readout in NB4 untreated (A) or ATRA treated cells (B) using PE-conjugated α-CD11c mAb; C and D) cell viability FACS readout in NB4 untreated (C) or ATRA treated cells (D) using propidium iodine. [file 1756-0500-3-24-S4.PDF]

**NB4**

**UT**

**T**

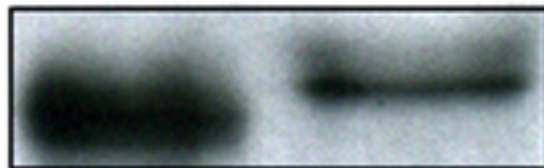

← **E2F1**

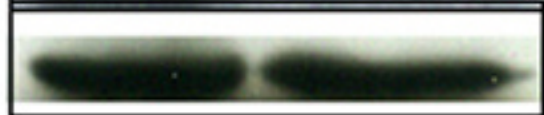

← **γ-tubulin**

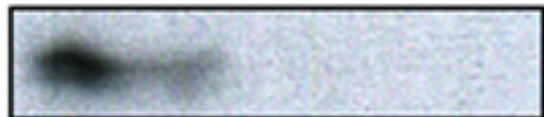

← **E2F4**

Supplement: Additional file 6 — E2F1 and E2F4 proteins level don't correlate with the miR-25 and miR-17 expression in NB4+ ATRA treated cells. 30 μg of total proteins were extracted at the indicated times from ATRA treated (T) or untreated (UT) NB4 cells and analyzed by western blot using α-E2F1 and α-E2F4 polyclonal antibodies (Santa Cruz). Input proteins were equalized by detecting the endogenous γ-tubulin. [file 1756-0500-3-24-S6.PDF]
