# Supplementary material for: Non-coding RNAs change their expression profile after Retinoid induced differentiation of the promyelocytic cell line NB4
Source: BMC Res Notes. 2010 Jan 27;3:24. doi: 10.1186/1756-0500-3-24 (PMC2843733; doi:10.1186/1756-0500-3-24)
Supplement: Additional file 3 — Additional M&M information. Materials and Methods for Array Fabrication, Fluorescent Labeling and Hybridization, Image Analysis and Data Collection, Human genome bioinformatic analysis, Microarray analysis, ATRA-induced differentiation of promyelocytic NB4 cells. Western blot analysis and Primers sequences for Real Time validation. [file 1756-0500-3-24-S3.DOC]

**Additional file 3**

**Title: Additional M&M information.**

Description: **Array Fabrication.**

496 50-mer amino-modified oligonucelotides were spotted in duplicate onto epoxy slides (Schott Nexterion) with SpotArray 24 (Perkin Elmer) at a 50 µM concentration in 3X SSC. A complete annotated list of these oligo is available(supplementary material). The post printing treatment was performed according to manufacturer. Briefly, after printing slides was incubated in humidity chambers (>90% relative humidity) at room temperature for 30 min for completion of the covalent binding and then incubated at 60°C for 30 min for immobilization of amino-modified oligos. The slides was stored dust-free, protected from light and under dry conditions upon use.

**Fluorescent Labeling and Hybridization.**

The hybridization method used in this work follows the Dual chip method and protocols (Eppendorf, Hamburg, Germany) [www.eppendorf.com/microarrays](http://www.eppendorf.com/microarrays). Each subarray in a slide was hybridized with one RNA sample (single labeling experiment and analysis of absolute fluorescence values) and compared with the control RNA in the second subarray. Each experiment was performed at least in three replicates (using 10 µg of total RNA/experiment, according to the RNA amounts available). A reference RNA sample composed of a pool of tissues (Stratagene) was used throughout all of the hybridizations to ensure normalized measures for each gene in each individual sample. Labelling of total RNA was achieved by direct incorporation of Cy5-dUTP or Cy3-dUTP (Amersham Bioscience) in a reverse transcription reaction using anchored oligodT primer (Invitrogen) and Superscript III reverse transcriptase (Invitrogen) according to manufacturer instructions. Fluor-tagged cDNAs were purified, dried by vacuum, then resuspended in the hybridization buffer (3X SSC, 50% formamide, 0,1%SDS).

Slides was activated in 0,1%Triton X-100 for 5’, then rinsed 2 times in 1 mM HCl for 2 min, rinsed in 100 mM KCl for 10 min and finally washed in dH_2_O at room temperature. After washing the blocking was performed incubating the slides in the blocking buffer (50 mM ethanolamine, 0,1% SDS in 0.1 M Tris pH 9) at 50°C for 15 min. Finally the slides were rinsed 1 min in dH_2_O at room temperature and centrifuged at 200g for 5 min to avoid water stains on the slide surface.

Hybridization was performed overnight, at 50 ºC in a humidity controlled chamber. A wash step in 2X SSC and 0.2% SDS was performed at 50° for 10 min, followed by a wash in 2X SSC for 10 min at room temperature, a final wash in 0.2X SSC for 10 min at room temperature and a centrifugation at 200g for 5 min to avoid any water stains on the slide surfaces.

**Image Analysis and Data Collection.**

Hybridized slides were scanned using the Affymetrix 428 array scanner. Data from the laser scans were collected as graphics (.tiff format) for each channel. The images were combined and processed using the ScanAnalyze program (written by Michael Eisen, <http://rana.Stanford.EDU/software/>) and the positions on the images were assigned to grid coordinates for the matching of spot identity with average pixel intensity at that region. The background was determined from pixel intensity at the edge of the bounding box. Background corrected, log converted intensity ratios were determined for each element of the array. The image processing analysis now also extracts information regarding spot quality and assigns a quality score to each ratio measurement. The definition of the quality metric is based on the notion that unreliable data points usually result from weak target intensity, high local background, small target area, and inconsistent target intensity within a given target. Implementation of the quality metric enables unified and universally applicable data filtering before downstream higher-level data analysis.

ScanAlyze data was imported in GeneSpring for further elaboration (Silicon Genetics, Redwood City, CA). To import data it a file for each sample containing information about its gene name, signal and detection for each gene, was created. After importing data several experiments defining parameters and normalization were tested. Parameters are not numerical but define the sample nature or tissue type. The normalization of each sample was performed dividing each signal intensity by the 50^th^- percentile of all genes in that array. The normalization of each gene between samples was performed on the median of signal intensity of that gene in the reference sample.

By means of GeneSpring genes were analyzed for differential expression level between sample classes. The software allow the data filtering upon signal intensity and detection value. To select differentially expressed genes we used several approaches: for two class matching we utilized the Scatter Plot selecting the genes with an expression fold >2. To identify differentially expressed genes in a multi class match we calculated the log2 of normalized data. In this way over-expressed genes was the ones with a value >0, while the down-expressed was the ones with a value <0. For statistical validation we used t-test with a p-value of 0.01

**Human genome bioinformatic analysis**

Long non-protein coding RNA genes were selected from the annotated genes on human genome, as H-Invitational Database 5.0 (H‑invDB) and ncRNA databases. Long non-coding RNAs containing exon-intron structure, multi-exonic structure and located distant from other transcription units on the chromosome were named large intervening non-coding RNAs (lincRNAs). In mammals, several hundreds of lincRNAs were characterised in cell cultures. The npcRNAs were analyzed bioinformatically taking into account the evolution of the annotations of the human and mouse genome (in particular the possible extension of the annotated 5’ and 3’ gene extremities). This analysis showed that one hundred npcRNA candidates could be UTR sequences of the neighbouring gene on the genome browser at UCLA Santa Cruz, validating 330 candidates as independent npcRNAs, possessing very limited ORF regions. In conclusion, in this ribochip 330 npcRNA candidates represent different classes of RNAs, someone being potentially a functional lincRNA, while no antisense RNA was chosen in this study. We selected two different oligonucleotides for identification of SRA and XIST, while the oligonucleotide capture probe for Malat-1 was put in this set although it cannot be detected because it is processed in the nuclei in a longer speckle-associated RNA and a short cytoplasmic RNA.

**Microarray analysis**

We selected 492 human genes to design 50-mer oligonucleotide probes. 330 genes were human putative npcRNA candidates and a series of known cancer-related npcRNAs (SRA, Malat-1, PRINS, DLEU-1, H19, SRA, HIS-1, NTT, NSCLC, PCGEM1 and DD3). 150 genes were low abundance transcripts classified as coding for hypothetical proteins, not contained in the standard 25K commercial DNA microarrays, in addition to house-keeping genes and few non-human negative control probes.

**ATRA-induced differentiation of promyelocytic NB4 cells.**

The differentiation protocols of NB4 cells upon treatment with retinoids are well established. It has been shown that under continuous treatment with 1µM *All Trans Retinoic Acid* (ATRA), proliferation of NB4 cells is no longer detectable after three days and after five days the cells are functionally and morphologically similar to terminally differentiated polynuclear neutrophils (Lanotte et al. 1991). Nevertheless, in this work the protocol was firstly standardized taking into account that ATRA treatment may also lead to cell death thus disturbing the correct interpretation of the results. Cells were treated with different concentrations of ATRA (from 0.25 µM to 2 µM) and samples were harvested at different time points. The differentiation of the promyelocytic cells into granulocytes was profiled by FACS analysis measuring the number of cells expressing CD11c^+^, a marker of granulocyte differentiation. After 6 days of 0.5 µM ATRA treatment, we observed the highest number of differentiated cell, without a significant increase in dead cells compared to the mock treated cells. Briefly, 88% ± 6% CD11c^+^ and 1.5% ± 0.3% CD11c^+^ in treated versus untreated cells respectively (Figure 1 A and B), and 6% ± 2% dead 1.2% ± 0.2% dead respectively (Figure 1 C and D), as average of five independent experiments (see Figure 1). All following experiments were performed in the same conditions after monitoring CD11c^+^ levels and cell viability.

**Western blot analysis.**

1x10^7^ cells were harvested at various time of ATRA or mock treatment, centrifuged and washed with PBS. The cell pellets were resuspended in 1 ml of lysis buffer (10 mM Hepes pH 7.4, 150 mM KCl, 1 mM EDTA pH8, 1% Triton X-100, 1 mM DTT, 1mM orthovanadate, 1 mM NaF and protease inhibitor cocktail [Roche]) at 4° C for 20’. Samples were resuspended in Laemmli buffer and resolved by SDS-PAGE on 12% acrylamide/bis-acrylamide running gel. The proteins were electro-blotted on nitrocellulose membrane (Protran, Schleicher and Schuell), blocked in 5% of “non fat milk” (BioRad) 0.5 % BSA in PBS for two hrs. at room temperature and immunoblotted with pAb against E2F1 or E2F4 or mAb against γ-Tubulin (Sigma). Secondary antibodies (Sigma), horse-radish peroxidase conjugated, were used for detection by enhanced chemiluminescence (ECL, Amersham) followed by autoradiography (Hyperfilm, Amersham) and densitometric analysis performed by Image-J software.

**Primers sequences for Real Time validation**

THC1225071

Fw: tcttcactgggaggcttgtt

Rev. ccttgaagtggcagagaagg

AK021516

Fw: cctcctctcacttgctggtc

Rev: gggttaaatgtgaggccaga

nr_002196_H19

Fw: tcaagacaccatcggaaca

Rev: gctcagctctgggatgatgt

ak022994

fw: gagtgagcatgggagtggat

rev: caggttttacgggtcaggaa

ak092435

fw: atctccccctcctcctacct

rev: aggggaacggagagagaaaa

ak128567

fw: tcttcaggtggacaatgctg

rev: ggcaggaaaagtgcaaatgt

ak027352

fw: ggtggcttcttcttgtttgc

rev: ttacgatgcccacccttaac

ak097934

fw: tctgctcctccttcaccttc

rev: caggagggggatcagctact

al122122

fw: AtCACACCAACCCTCAAAGC

rev: TCCACCAGATCTCCATCTCC

cr593144

fw: ctgtcctgtgctgtgtgacc

rev: gggctctcagccatcatcta

thc1242508

fw: GACTGCATTTGCTTGCCTTC

rev: GGTGGCTTCAGGCAAATAAG
