# Supplementary material for: Non-coding RNAs change their expression profile after Retinoid induced differentiation of the promyelocytic cell line NB4
Source: BMC Res Notes. 2010 Jan 27;3:24. doi: 10.1186/1756-0500-3-24 (PMC2843733; doi:10.1186/1756-0500-3-24)
Supplement: Additional file 5 — miRNAs equally expressed in both ATRA treated and untreated NB4 cells by microarray analysis. Hsa = human miRNAs; mmu = miRNAs [file 1756-0500-3-24-S5.PDF]

1 hsa-let-7b-prec
2 hsa-let-7c-prec
3 hsa-let-7d-prec
4 hsa-let-7d-v2-precNo2
5 hsa-let-7e-prec
6 hsa-mir-007-3-precNo1
7 hsa-mir-009-1No1
8 hsa-mir-023a-prec
9 hsa-mir-023b-prec
10 hsa-mir-024-prec
11 hsa-mir-026a-precNo1
12 hsa-mir-026b-prec
13 hsa-mir-032-precNo1
14 hsa-mir-032-precNo2
15 hsa-mir-033b-prec
16 hsa-mir-034precNo1
17 hsa-mir-092-prec-13=092-1No1
18 hsa-mir-092-prec-13=092-1No2
19 hsa-mir-092-prec-X=092-2
20 hsa-mir-093-prec-7.1=093-1
21 hsa-mir-103-2-prec
22 hsa-mir-103-prec-5=103-1
23 hsa-mir-107-prec-10
24 hsa-mir-122a-prec
25 hsa-mir-128a-precNo2
26 hsa-mir-146-prec
27 hsa-mir-147-prec
28 hsa-mir-150-prec
29 hsa-mir-184-precNo2
30 hsa-mir-185-precNo1
31 hsa-mir-185-precNo2
32 hsa-mir-191-prec
33 hsa-mir-192-2/3No1
34 hsa-mir-192-precNo2
35 hsa-mir-194-prec
36 hsa-mir-196-1-precNo1
37 hsa-mir-196-2-precNo2
38 hsa-mir-197-prec
39 hsa-mir-198-prec
40 hsa-mir-202-prec
41 hsa-mir-206-precNo1
42 hsa-mir-210-prec
43 hsa-mir-212-precNo1
44 hsa-mir-213-precNo1
45 hsa-mir-214-prec
46 hsa-mir-220-prec
47 hsa-mir-221-prec
48 hsa-mir-222-precNo1
49 mmu-let-7i-prec
50 mmu-mir-023b-prec
51 mmu-mir-030e-prec
52 mmu-mir-099a-prec
53 mmu-mir-128-precNo1
54 mmu-mir-129-prec
55 mmu-mir-135-prec
56 mmu-mir-136-prec
58 mmu-mir-143-prec
59 mmu-mir-146-prec
60 mmu-mir-154-prec
61 mmu-mir-181-prec
62 mmu-mir-185-prec
63 mmu-mir-191-prec
64 mmu-mir-202-prec
65 mmu-mir-206-prec
66 mmu-mir-207-prec
67 mmu-mir-212-precNo1
68 mmu-mir-212-precNo2
69 mmu-mir-213-prec
70 mmu-mir-214-prec
71 mmu-mir-221-precNo1
72 mmu-mir-222-prec
